# On the possible role of ERK, p38 and CaMKII in the regulation of CGRP expression in morphine-tolerant rats

**DOI:** 10.1186/1744-8069-7-68

**Published:** 2011-09-21

**Authors:** Zhiyong Wang, Jean-Guy Chabot, Remi Quirion

**Affiliations:** 1 Dept. of Psychiatry, Douglas Mental Health University Institute, McGill University, Montreal, Quebec, H4H 1R3, Canada

**Keywords:** CGRP, ERK, p38, CaMKII, morphine

## Abstract

**Background:**

The neuropeptide, calcitonin gene-related peptide (CGRP) has been proposed to be a regulator of the development of morphine analgesic tolerance and thereby could be a target to reduce the induction of this phenomenon under clinical conditions. However, the mechanisms of CGRP regulation are unclear. We investigated here the possible role of the extracellular signal-regulated protein kinase (ERK), p38 and calcium/calmodulin-dependent protein kinase II (CaMKII) in CGRP regulation following chronic morphine treatment.

**Results:**

A 7-day treatment with morphine (15 μg/day) led to an increase in CGRP contents in the spinal cord dorsal horn (SCDH) and dorsal root ganglion (DRG) and this effect was prevented by the inhibition of the ERK, p38 or CaMKII pathway. The phosphorylation/activation of ERK, p38 and CaMKII was enhanced in the SCDH following chronic morphine while in DRG only the phosphorylation of CaMKII was increased. Moreover, our chronic morphine treatment up-regulated neuronal nitric oxide synthase (nNOS) levels in the SCDH, an effect blocked by the inhibition of the ERK, p38 or CaMKII pathway. The blockade of nNOS activity also suppressed chronic morphine-induced CGRP increases in the DRG and SCDH. Double immunofluorescence studies revealed that nNOS and CaMKII are co-localized in the SCDH and that CaMKII is activated in CGRP-expressing DRG neurons.

**Conclusions:**

The activation of spinal ERK, p38 and CaMKII, alongside nNOS, is involved in chronic morphine-induced CGRP up-regulation in both the DRG and SCDH. Moreover, the stimulation of CaMKII in the DRG likely directly regulates the expression of CGRP associated with morphine analgesic tolerance.

## Background

Opiates such as morphine are the most commonly used drugs in the clinical management of moderate to severe pain, including cancer pain. However, their clinical usefulness is largely hindered by the development of analgesic tolerance, which often requires escalating doses to achieve equivalent pain relief [1]. The mechanisms underlying this phenomenon have been extensively investigated and several hypotheses have been proposed, including the altered activity of excitatory substances and their intracellular signaling pathways, the desensitization of mu-opioid receptor and its possible linkage with arrestin as well as interaction between mu- and delta-opioid receptors [2]. In accordance with these data, a neuropeptide, calcitonin gene-related peptide (CGRP), has been suggested to play a major role in the development of tolerance to morphine-induced analgesia [3-7] and thus could be a promising target to reduce the occurrence of tolerance. Indeed, chronic morphine treatment results in an increase in CGRP expression and/or release in the spinal cord [3,5,6,8,9]. Moreover, treatment with a CGRP receptor antagonist was shown to prevent the development of tolerance to morphine-induced analgesia [3,4]. Furthermore, the role of CGRP in morphine tolerance may be attributable to its differential regulation of cell-type specific kinase-transcription factor cascades [5,6]. Accordingly, it is of interest to investigate how the expression of CGRP is regulated following chronic morphine treatment.

CGRP, a 37-amino acid neuropeptide is broadly distributed in the peripheral and central nervous systems, including the dorsal root ganglion (DRG) and its nerve terminals, which are the predominant source of CGRP in the spinal cord dorsal horn (SCDH) [10]. Mounting evidence has suggested that various factors influence CGRP expression under certain conditions. For example, CGRP levels can be increased in vivo or in vitro by growth factors such as nerve growth factor (NGF) or the cytokine activin A in sensory neurons [11-16]. In particular, peripheral stimulation such as inflammation can induce an increase in CGRP mRNA levels in the DRG, possibly through the synergistic effect of NGF and activin A [17]. Our previous results have also shown that chronic morphine-induced increases in CGRP levels may result from the activation of ERK and the downstream cAMP response element-binding protein (CREB) in cultured DRG sensory neurons [18]. In the present study, we investigated factors involved in the regulation of the expression of CGRP and associated with the development of tolerance to morphine-induced analgesia both at the level of the DRG and SCDH.

## Results

### Possible role of ERK, p38 and CaMKII in the development of morphine antinociceptive tolerance

**Figure 1:**
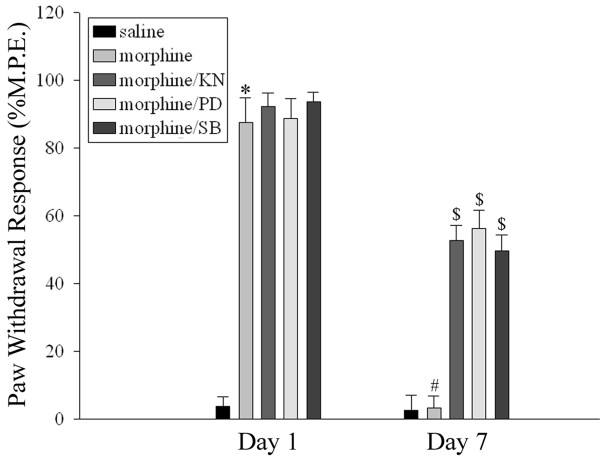
**Inhibition of the development of tolerance to morphine-induced analgesia by the co-administration of various inhibitors**. Paw-withdrawal latency was tested 30 min after morphine injection on days 1 and 7 following a daily intrathecal delivery of saline (10 μl), morphine (M, 15 μg/10 μl) alone or in combination with KN93 (KN, 15 nmol/10 μl), a CaMKII inhibitor, PD98059 (PD, 10 μg/10 μl), an MEK (ERK upstream kinase) inhibitor or SB203580 (SB, 10 μg/10 μl), a p38 inhibitor for 7 days. KN, PD and SB were administered 30 min before morphine injection. Data are expressed as mean ± SEM of 7-8 animals. *p < 0.01 *vs *saline group on day 1. #p < 0.01 *vs *morphine group on day1. $p < 0.01 *vs *morphine group on day 7.

**Figure 2:**
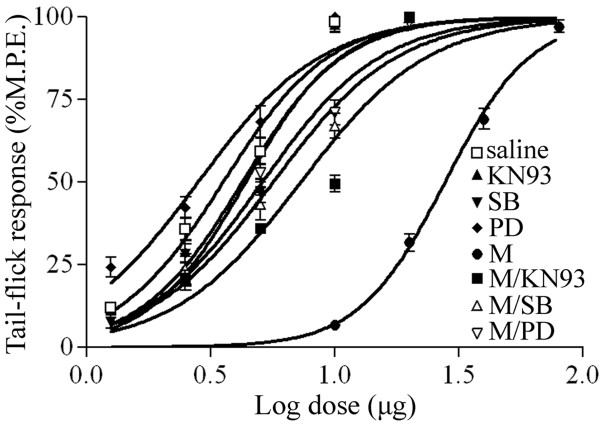
**Cumulative dose-response curve of the antinociceptive effects of acute intrathecal injection of morphine in animals previously receiving saline, KN93 (15 nmol), PD98059 (PD, 10 μg), SB203580 (SB, 10 μg), morphine (M) or morphine with KN93 (M/KN93, 15 nmol), PD98059 (M/PD, 10μg) or SB203580 (M/SB, 10μg)**. Data are expressed as mean ± SEM of 6 animals.

We have previously shown that the development of CGRP-associated tolerance to morphine-induced analgesia involves the activation of ERK, p38 and CaMKII [5,6]. As shown in Figure 1, an acute morphine treatment (15 μg) produced analgesia on day 1 as revealed by an increase in paw-withdrawal response. In contrast, a 7-day daily intrathecal delivery of morphine (15 μg/day) led to decreased paw-withdrawal responses. This effect was attenuated by a co-treatment with PD98059 (10 μg), a MEK (ERK upstream kinase) inhibitor, SB203580 (10 μg), a p38 inhibitor as well as KN93 (15 nmol), a CaMKII inhibitor (two way repeated ANOVA, F_(4,71) _= 68.877, p < 0.001). Furthermore, the 7-day treatment with morphine produced a shift in the dose-response curve, which was attenuated by the co-administration of PD98059 (10 μg), SB203580 (10 μg) or KN93 (15 nmol) (Figure 2) (one way ANOVA, F_(7,47) _= 253.198, p < 0.001). These inhibitors by themselves did not influence the shift in the dose-response curve when compared with the saline group (Figure 2).

### Involvement of ERK, p38 and CaMKII in CGRP regulation related to morphine antinociceptive tolerance

**Figure 3:**
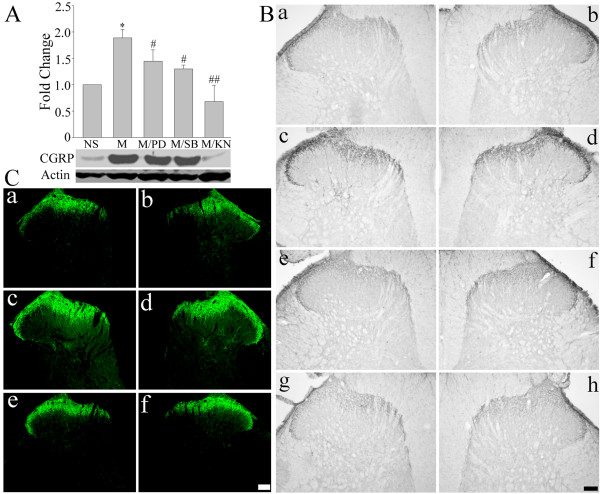
**Effects of the co-administration of various indicated inhibitors on chronic morphine-induced CGRP up-regulation in the spinal cord dorsal horn**. A) Western blot analysis revealed an increase in CGRP level following a daily intrathecal delivery of morphine (M, 15 μg/day) for 7 days, an effect prevented by a co-treatment with a MEK inhibitor, PD98059 (PD, 10 μg); a p38 inhibitor, SB203580 (SB, 10 μg); or a CaMKII inhibitor, KN93 (KN, 15 nmol). Data are expressed as mean ± SEM of 4 animals. *p < 0.01 *vs *saline (NS) group. #p < 0.05 *vs *morphine group. ##p < 0.01 *vs *morphine group. B and C) The immunohistochemical analysis showed that a 7-day treatment with morphine (15 μg/day) increased CGRP levels (Bc, d and Cc, d) when compared with the saline group (Ba, b and Ca, b), an effect inhibited by the co-administrationof PD (10 μg, Be, f), SB (10 μg, Bg, h) or KN (15 nmol, Ce, f). Scale bar, 200 μm (Ba-h, Ca-f).

**Figure 4:**
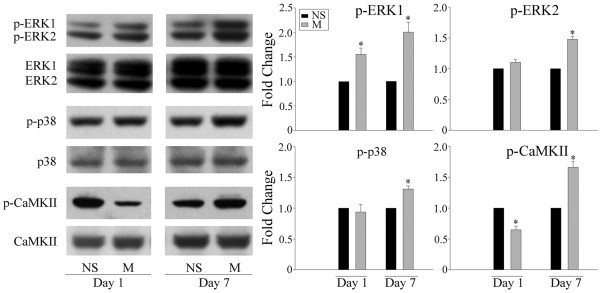
**Western blot analyses of the phosphorylation of ERK, p38 and CaMKII in the spinal cord dorsal horn following a chronic morphine treatment**. A 7-day treatment with morphine (M, 15 μg) increased the phosphorylated levels of ERK, p38 and CaMKII on day 7. Data are expressed as mean ± SEM of 4 animals. *p < 0.01 *vs *saline (NS) group.

Chronic morphine treatments have been shown to increase CGRP levels in primary afferent terminals of the spinal dorsal horn [3,5,6,8]. Accordingly, we investigated next if ERK, p38 and CaMKII are implicated in the regulation of CGRP expression following repeated treatments with morphine. As shown in Figure 3, a 7-day intrathecal delivery of morphine (15 μg/day) increased CGRP levels in the spinal cord dorsal horn, as revealed by western blot (Figure 3A; two way ANOVA, F_(4,19) _= 13.400, p < 0.001) and immunohistochemical (Figure 3B and 3C) analyses. This effect was inhibited by the co-administration of PD98059 (10 μg), SB203580 (10 μg) or KN93 (15 nmol). A 7-day daily injection of these inhibitors by themselves failed to alter CGRP levels (additional file 1, figure 1; one way ANOVA, F_(3,15) _= 0.916, p = 0.462). In addition, the levels of phosphorylated ERK (two way ANOVA, for p-ERK1 F_(3,15) _= 22.248, p < 0.001; for p-ERK2, F_(3,15) _= 34.437, p < 0.001), p38 (two way ANOVA, F_(3,15) _= 25.351, p < 0.001) and CaMKII (two way ANOVA, F_(3,15) _= 58.368, p < 0.001), indicative of their activation, were increased in the spinal dorsal horn following repeated morphine treatment (Figure 4).

**Figure 5:**
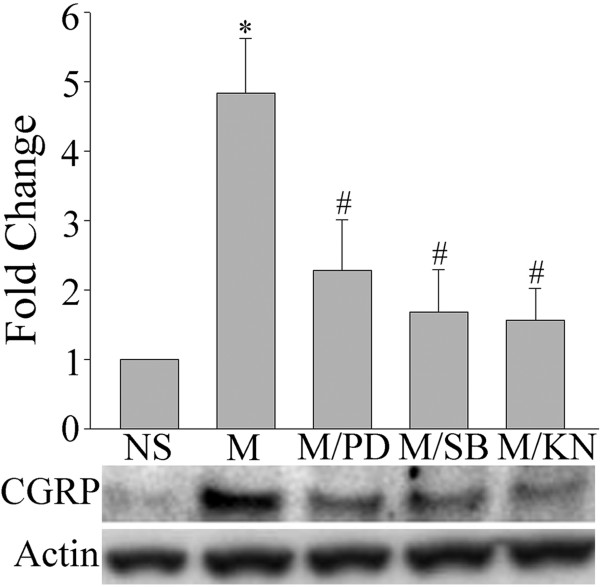
**Inhibition of chronic morphine-induced CGRP up-regulation by the co-administration of various inhibitors**. Western blot analyses revealed that a 7-day treatment with morphine (M, 15 μg) increases CGRP levels in the DRG. This effect was blocked by the co-delivery of PD98059 (MEK inhibitor, PD, 10 μg), SB203580 (p38 inhibitor, SB, 10 μg) or KN93 (CaMKII inhibitor, KN, 15 nmol). Data are expressed as mean ± SEM of 4 animals. *p < 0.01 *vs *saline (NS) group. #p < 0.01 *vs *morphine group.

**Figure 6:**
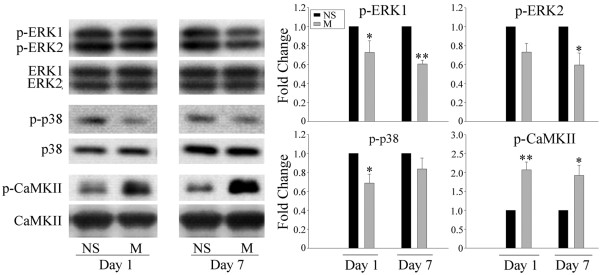
**Western blot analyses of the phosphorylation of ERK, p38 and CaMKII in the DRG following a chronic morphine treatment**. A 7-day treatment with morphine (M, 15 μg) increased the level of phosphorylated CaMKII, but not those of ERK or p38. Data are expressed as mean ± SEM of 4 animals. *p < 0.05 *vs *saline (NS) group. **p < 0.01 *vs *saline group.

**Figure 7:**
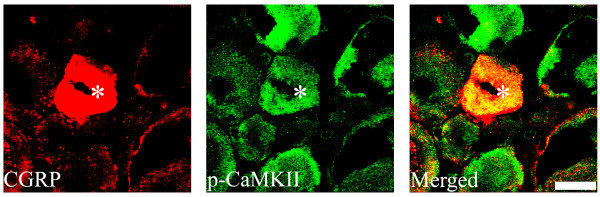
**Double immunofluorescence staining shows that phosphorylated CaMKII (p-CaMKII, green) is expressed in CGRP-expressing (red) DRG neurons**. Arrows show their co-localization (yellow; asterisk). Scale bar, 20 μm.

Since DRG neurons are the predominant source of CGRP in the spinal cord dorsal horn [10], we examined next the changes in CGRP expression at the DRG level. CGRP levels were up-regulated following a 7-day intrathecal injection of morphine (15 μg/day) (Figure 5, two way ANOVA, F_(4,19) _= 12.036, p < 0.001). This up-regulation was prevented by the inhibition of ERK (PD98059, 10 μg), p38 (SB203580, 10 μg) and CaMKII (15 nmol) pathways (Figure 5). Interestingly, the 7-day treatment with morphine did not increase the levels of phosphorylated ERK and p38, but indeed increased p-CaMKII level (Figure 6). The confocal study showed that CGRP is also present in p-CaMKII-expressing cells, suggesting their co-localization(Figure 7).

### Possible role of nNOS in the regulation of CGRP by ERK, p38 and CaMKII during the development of morphine tolerance

**Figure 8:**
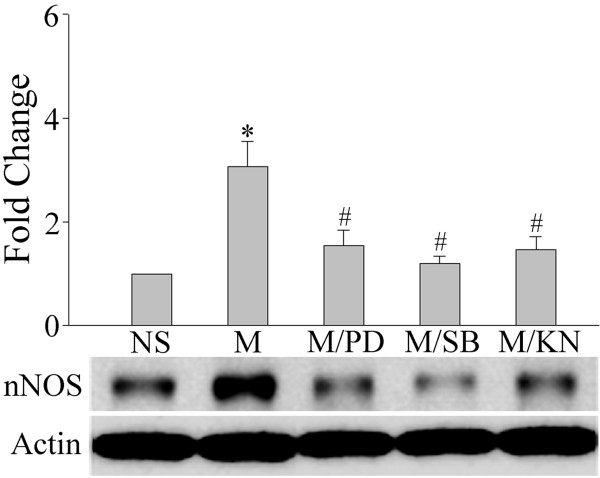
**Chronic morphine-induced increases in nNOS are inhibited by the co-administration of various inhibitors**. Western blot analyses revealed that a 7-day treatment with morphine (M, 15 μg) increased nNOS levels in the spinal cord dorsal horn, an effect inhibited by the co-delivery of PD98059 (MEK inhibitor, PD, 10 μg), SB203580 (p38 inhibitor, SB, 10 μg) or KN93 (CaMKII inhibitor, KN, 15 nmol). Data are expressed as mean ± SEM of 4 animals. *p < 0.01 *vs *saline (NS) group. #p < 0.01 *vs *morphine group.

**Figure 9:**
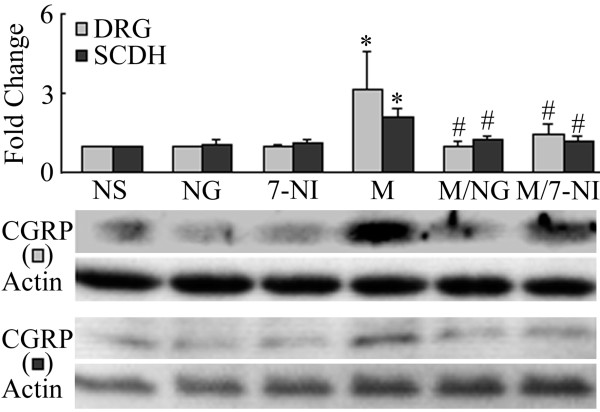
**Western blot analyses of CGRP levels in the DRG and spinal cord dorsal horn (SCDH) following chronic treatments with saline (NS), NG (a nNOS inhibitor, 75 μg), 7-NI (an nNOS inhibitor, 25 μg) and morphine (M, 10 μg) alone or in combination with NG (75 μg) or 7-NI (25 μg)**. A 7-day treatment with morphine increased CGRP levels in both the DRG and SCDH, effects inhibited by the co-administration of the nNOS inhibitors NG and 7-NI. *p < 0.01 *vs *respective saline group. #p < 0.01 *vs *respective morphine group.

**Figure 10:**
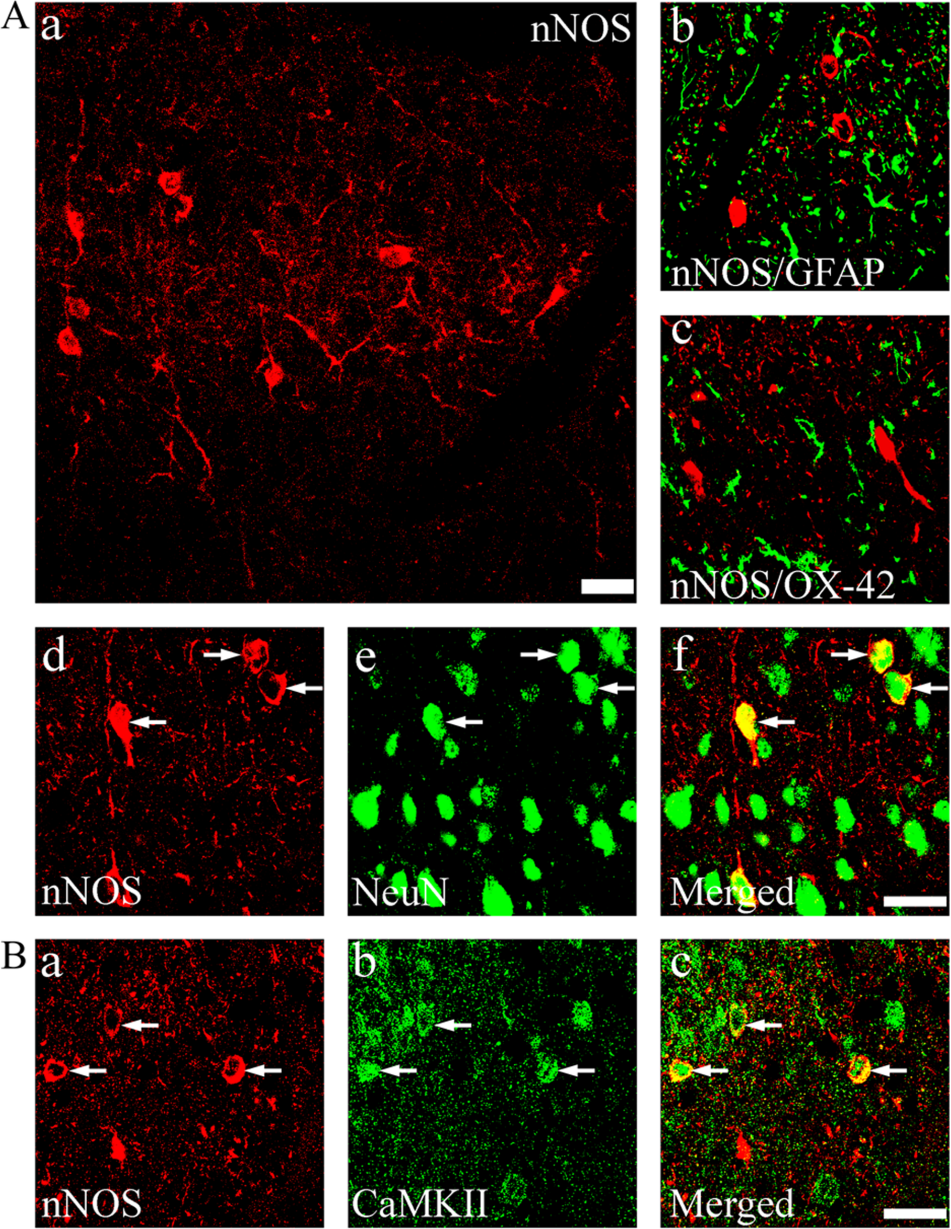
**Immunohistochemical staining and distribution of nNOS-ir cells in the spinal cord dorsal horn (Aa)**. Double immunofluorescence staining revealed that nNOS is mostly enriched in NeuN-ir (arrows, Ad-f,) but not in GFAP- (green, Ab) or OX-42-ir (green, Ac) cells. B) nNOS-expressing cells also express CaMKII (arrows, Ba-c). Scale bars, 25 μm (Aa); 20 μm (Ab-f, Ba-c).

It has been proposed that spinal nitric oxide (NO) can act as a retrograde signaling molecule to influence CGRP release from presynaptic primary afferent terminals in the spinal dorsal horn [19]. To investigate the possible role of NO in the regulation of CGRP expression in this region, we used (4S)-N-(4-Amino-5[aminoethyl]aminopentyl)-N'-nitroguanidine, TFA (NG) and 7-Nitroindazole (7-NI) to specifically inhibit neuronal NO synthase (nNOS). We first determined the nNOS levels following a chronic morphine treatment. A repeated treatment with morphine (15 μg/day) for 7 days markedly increased nNOS expression (Figure 8, two way ANOVA, F_(4, 19) _= 17.471, p < 0.001) while inducible NOS (iNOS) and endothelial NOS (eNOS) levels were not changed (data not shown). This increase was inhibited by a co-treatment with PD9059 (10 μg), SB203580 (10 μg) or KN93 (15 nmol), suggesting a role for these kinases in our model (Figure 8). In contrast, a 7-day treatment with these kinase inhibitors alone did not significantly alter nNOS levels (additional file 1, figure 1; F_(3,15) _= 2.893, p = 0.094). We then examined if the inhibition of nNOS activity affected CGRP expression in the SCDH and DRG. As shown in Figure 9, a co-treatment with the nNOS inhibitors NG (75 μg) or 7NI (25 μg) prevented chronic morphine-induced increase in CGRP levels both in the SCDH (two way ANOVA, F_(5,23) _= 7.304, p = 0.001) and DRG (two way ANOVA, F_(5,23) _= 5.071, p = 0.006) while NG (75 μg) or 7NI (25 μg) alone did not change CGRP levels in both of these two regions (Figure 9). nNOS is mostly enriched in neurons of the spinal dorsal horn, but not in microglia or astrocytes (Figure 10A). CaMKII was also predominantly seen in neurons (additional file 1, figure 2). Furthermore, CaMKII was found to be localized in nNOS-expressing cells (Figure 10B), suggesting their co-localization. Finally, we also examined possible changes in nNOS levels in the DRG and no differences were observed (data not shown).

## Discussion

In the present study, we investigated intracellular signaling molecule(s) involved in the regulation of CGRP known to participate in the development of tolerance to morphine's analgesia. Our data suggest a role of ERK, p38 and CaMKII, as shown by enhanced activation/phosphorylation of these kinases in the SCDH while their inhibitions prevented chronic morphine-induced CGRP increases as well as the development of tolerance to morphine-induced analgesia. The CGRP-related regulatory processes may involve retrograde signaling via the NO signal transduction system because the level of nNOS, a major NO-synthesizing enzyme, is increased in the SCDH following chronic morphine treatment and can be modulated by the inhibition of either ERK or p38 or CaMKII pathway. Moreover, the blockade of nNOS also inhibited chronic morphine-induced increases in CGRP level. The morphological data showed that CaMKII and nNOS are co-localized in neurons of the SCDH. In the DRG, increased CaMKII activation/phosphorylation was observed in CGRP-expressing neurons in morphine-tolerant animals. Taken together, chronic morphine-induced CGRP up-regulation in both the DRG and SCDH likely involves the activation of spinal ERK, p38 and CaMKII as signaling molecules, a process also requiring nNOS. Additionally, the activation of CaMKII in the DRG may directly influence the expression of CGRP required for the development of tolerance to morphine-induced analgesia.

The neuropeptide CGRP has been proposed to play an important role in spinal nociceptive processing [20,21]. Once released, CGRP can act both pre- and post-synaptically on functional CLR/RAMP1 family 3 GPCR receptors [22], leading to the activation of various downstream signaling molecules including protein kinase A (PKA), PKC, CaMKII and MAP kinase involved in the multiple and complex pathophysiological effects induced by CGRP [22-24]. We have previously demonstrated a major role of CGRP and its receptors in the pathogenesis of morphine antinociceptive tolerance [3-6]. Specifically, blockade of CGRP receptors using peptide (CGRP8-37) as well as non-peptide (BIBN4096BS) CGRP receptor antagonists prevented the development of tolerance to morphine-induced analgesia as well as chronic morphine-induced CGRP up-regulation in the DRG and SCDH [3-6]. Several kinases including ERK, p38 and CaMKII have also been suggested to be involved in the development of tolerance to morphine-induced analgesia [5,6,25,26] and their activation can be regulated by the blockade of CGRP receptor signaling [5,6]. Therefore it was deemed to be of major interest to investigate the link between CGRP expression/regulation and kinase activities in the development of tolerance to morphine-induced analgesia. Accordingly, we examined the activity of the aforementioned kinases in both DRG and SCDH in tolerant animals. In agreement with earlier studies [5,6,25,26], ERK, p38 and CaMKII showed increased levels in their phosphorylation/activation in the SCDH. We then used specific inhibitors to block each of the three kinase pathways. These various inhibitors blocked chronic morphine-induced CGRP increases in both DRG and SCDH. These data strongly suggest an existence of a key relationship between CGRP expression/regulation and these kinases in the development of tolerance to morphine-induced analgesia.

It is well established that CGRP is predominantly derived from nerve terminals of the DRG primary afferent fibers in the SCDH [10]. Available data have also shown that activated/phosphorylated ERK and p38 kinases are expressed in glial cells in the SCDH [5-7,26] while activated CaMKII is mostly present in neurons [6]. Hence, how is it that messengers expressed in both neurons and glia can have similar effects on the modulation of CGRP observed in morphine tolerant animals? It is known that as a diffusible molecule, NO plays a crucial role in the development of morphine analgesic tolerance [27,28]. Once released, NO can exert its effects either via the activation of the soluble guanylate cyclase (sGC)-cyclic GMP (cGMP) pathway [29,30] or directly by modulating gene expression through its S-nitrosylation of target proteins [31]. Therefore, we hypothesize that NO can act as an intermediate linking the activation of kinases to CGRP expression during the development of tolerance to morphine-induced analgesia. In support of this hypothesis, our data have demonstrated significant increases in nNOS levels in the SCDH in morphine-tolerant animals. These increases can be prevented by the inhibition of the ERK, p38 and CaMKII pathways. In addition, both CaMKII and nNOS are co-localized, suggesting the existence of a CaMKII-nNOS cascade regulating CGRP expression following chronic morphine treatment. As mentioned earlier, both ERK and p38 are mostly expressed in glial cells, suggesting an indirect effect by the modulation of these kinases. Interestingly, the inhibition of nNOS activity largely blocks chronic morphine-induced CGRP up-regulation in the DRG and SCDH, indicating that NO may act as a retrograde signaling molecule to regulate presynaptic CGRP expression levels. Taken together, these data suggest that chronic morphine-induced CGRP up-regulation involves the activation of a neuronal CaMKII-nNOS pathway, a process that can be regulated by the activation of ERK and p38 via a glial-neuronal cell interaction.

It should be noted that the western blot analyses did not reveal any increases in the phosphorylation of ERK and p38 in DRG extracts. In contrast, a chronic morphine treatment increased ERK phosphorylation in cultured DRG [18] as well as the phosphorylation of ERK and p38 in vivo [32]. However, CaMKII showed a significant increase in its phosphorylation in the DRG, suggesting different molecular responsiveness of these kinases to morphine exposure and the development of tolerance. The different molecular responses could be attributable to the differential cell localization of kinases in the two regions, i.e. glia-localized in spinal cord and neuron-localized in DRG. In addition, activated CaMKII was found to be expressed in CGRP-enriched DRG neurons with the inhibition of CaMKII activity decreasing CGRP expression, suggesting that the chronic morphine-induced CGRP up-regulation may also involve the direct activation of CaMKII in DRG neurons.

**Figure 11:**
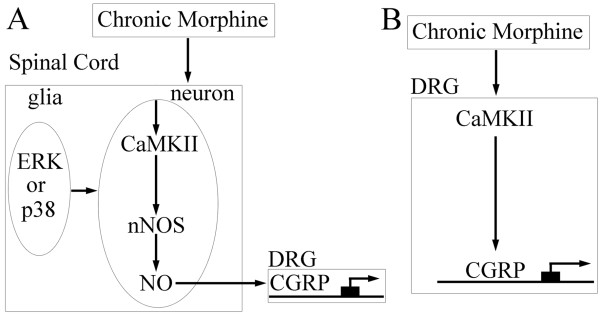
**Schematic models of the mechanisms underlying the regulation of CGRP during the development of tolerance to morphine-induced analgesia**. A chronic morphine treatment induces the activation of the neuronal CaMKII-nNOS pathway in the SCDH, resulting in an increased NO release. NO can then retrogradly diffuse to the presynaptic nerve terminals to modulate CGRP gene expression either via direct activation of the sGC-cGMP pathway [29,30] or via the S-nitrosylation [31] of related nuclear proteins. The effects of glial ERK and p38 on CGRP expression in the SCDH may occur through the regulation of neuronal CaMKII-nNOS signaling. B) In the DRG, chronic morphine enhances the activation of CaMKII, which can then stimulate CGRP gene expression via the modulation of various transcriptional factors.

In summary, a chronic morphine treatment induced the activation of the neuronal CaMKII-nNOS pathway in the SCDH, thereby resulting in increased NO release. The released NO diffuses to presynaptic nerve terminals to retrogradely modulate CGRP gene expression via the direct activation of the sGC-cGMP pathway [29,30] or the S-nitrosylation [31] of related nuclear proteins (Figure 11A). The effects of glial ERK and p38 [5,6,26] on the expression of CGRP in the SCDH likely occur through the regulation of neuronal CaMKII-nNOS signaling (Figure 11B). In the DRG, a chronic morphine treatment can enhance CaMKII activation resulting in CGRP gene expression via the modulation of transcriptional activities leading to tolerance to opiate-induced analgesia. Accordingly, CGRP likely has a key role in the development of tolerance and blockers of the pathways and/or molecules involved could prove most useful in various pathophysiological conditions.

## Methods

All animal experiments were approved by the Animal Care and Use Committee of McGill University as well as Canadian Council on Animal Care and performed according to the guidelines of the International Association for the Study of Pain concerning the use of laboratory animals.

### Operative procedures and drug delivery

Intrathecal catheterization was performed on male Sprague-Dawley rats weighing 230-250 g as previously described [5-7]. Specifically, under isoflurane anesthesia, a polyethylene (PE)-10 tubing was implanted into the subarachnoid space through the gap between the vertebrae L5 and L6 and extended to the caudal part of the lumbar enlargement. The remaining portion of the tubing was partly sutured to the neighboring musculature and exited through a small hole at the back of the neck. Animals were allowed to recover for 7-9 days before behavioral experiments and were acclimated to the testing environment. Drugs were injected through the exterior end of the tubing in a total volume of 10 μl followed by a flush of 15 μl of saline. Drug doses were chosen in accordance with previous studies [3,5,6,26,33]. The following drugs were purchased from Sigma-Aldrich (St. Louis, MO, USA): morphine sulfate (1.5 μg/μl in saline), 4-(4-fluorophenyl)-2-(4-methylsulfinylphenyl)-5-(4-pyridyl)1H-imidazole (SB203580; 1 μg/μl in 20% DMSO) and 2'-amino-3'-methoxyflavone (PD98059; 1 μg/μl in 20% DMSO) while 2-[*N*-(2-hydroxyethyl)]-*N*-(4-methoxybenzenesulfonyl)]amino-*N*-(4-chlorocinnamyl)-*N*-methylbenzylamine)phosphate (KN93; 1.5 mM in 20% DMSO), 7-Nitroindazole (7-NI; 2.5 μg/μl in 20% DMSO) and (4S)-N-(4-amino-5[aminoethyl]aminopentyl)-N'-nitroguanidine, TFA (NG; 7.5 μg/μl in 20% DMSO) were purchased from Calbiochem (San Diego, CA, USA).

### Behavioral testing

The standard regimen of daily intrathecal injection of morphine (15 μg/day for 7 days) was used to induce analgesic tolerance to morphine as previously described [3,5,34]. Analgesic effects of morphine were evaluated by paw-withdrawal latency. Specifically, a radiant heat beam was focused on the plantar surface of the hindpaw [35]. The thermal stimulation intensity was adjusted to produce a baseline latency of 10-12 sec and a cut-off time of 25 sec was used to prevent any potential tissue injury. Paw-withdrawal response latency was defined as the time from the onset of radiant heat to paw withdrawal and measured automatically by a digital meter. We conducted behavioral testing before and 30 min after drug injection on days 1 and 7. Data were standardized by conversion to percentage of maximal possible effect (%MPE) according to the following formula: [(response latency-baseline latency)/(cut-off time-baseline latency)]×100.

On day 8 ascending doses of morphine were acutely injected to animals previously receiving various chronic treatments through the intrathecal catheter every 30 min until a maximal analgesic effect was achieved in the tail-flick test, as previously reported [3,6]. The cumulative dose-response curve of the antinociceptive effect of acute morphine was constructed accordingly. A non-linear regression analysis was used to determine the ED_50 _values while their statistical significance was computed using one-way ANOVA followed by a student-Newman-Keuls (SNK) *post-hoc *test for comparisons between groups. The standardized behavioral data were expressed as mean ± SEM and analyzed by two-way repeated ANOVA with a SNK *post hoc *test.

### Immunohistochemistry

Animals were perfused via the ascending aorta with 300 ml of 4% paraformaldehyde following a flush of 100-150 ml saline. The lumbar spinal cords (L4 and 5) and corresponding DRGs were harvested, post-fixed in the same fixative for 4 h at 4°C and then transferred to 30% sucrose overnight for cryoprotection. DRG (16 μm) and transverse spinal cord sections (35 μm) were cut on a cryostat and collected for immunohistochemical studies.

#### Single labeling of CGRP

To remove endogenous peroxidase activity, floating spinal cord sections were treated with 0.03% H_2_O_2 _for 15 min. Next, to block any non-specific binding and enhance membrane permeability, sections were processed with 10% normal donkey serum and 0.03% TrionX-100 in 0.01 M PBS at room temperature (RT) for 30 min. Sections were incubated at 4°C with the primary guinea pig anti-CGRP antibody (1:3000, Peninsula Labs, San Carlos, CA, USA) for 48 hr. After rinsing in 0.01 M PBS, sections were incubated at RT in biotinylated donkey anti-guinea pig IgG for 1 h followed by reaction with avidin-biotin-peroxidase complex (ABC) for 30 min using Vectastain ABC kit (Vector, Burlingame, CA, USA). The immunoprecipitates were developed in 0.05% diaminobenzidine with 0.01% hydrogen peroxide and intensified by the addition of 0.02% nickel ammonium sulfate. In addition, CGRP fluorescent staining (1:500) was performed with the same primary antibody and visualized using Alexa488-conjugated donkey anti-guinea pig secondary antibody.

#### Double immunolabeling

To reveal if CaM kinase II (CaMKII) can be activated in CGRP-expressing neurons, DRG sections were incubated with a mixture of rabbit anti-p-CaMKII (1:400) and guinea pig anti-CGRP (1:500). To identify the cell type(s) expressing nNOS or CaMKII, spinal sections were incubated with a mixture of rabbit anti-nNOS (or rabbit anti-CaMKII) and mouse anti-glial fibrillary acidic protein (GFAP, an astroglial marker, 1:1000, Millipore, Billerica, MA, USA), or mouse anti-OX-42 (a microglial marker, 1:100, AbD Serotech, Raleigh, NC, USA) or mouse anti-NeuN (a neuronal marker, 1:1000, Millipore) antibodies. Spinal sections were also incubated with a mixture of goat anti-nNOS and rabbit anti-CaMKII to determine if these two molecules are co-localized.

### Western blotting

Animals were rapidly perfused through the ascending aorta with 100-150 ml of saline under anesthesia and the L4-L6 spinal cords and corresponding DRGs were collected on dry ice and stored at -80°C. Tissues were homogenized in modified RIPA buffer (50 mM Tris-HCl, 150 mM NaCl, 1 mM EDTA, 1% Igepal CA-630, 0.1% SDS, 50 mM NaF, and 1 mM NaVO_3_) containing a mixture of protein inhibitors (Sigma) including PMSF (2 mM), leupeptin (10 μg/ml) as well as aprotinin (10 μg/ml) and protein concentrations were determined by using a BCA protein assay kit (Pierce Biotechnology, Rockford, IL, USA). Equal amounts of protein samples (20 μg/lane) were loaded and separated on SDS-PAGE gels (4-20% tris-glycine gels, Invitrogen, Carlsbad, CA, USA; for CGRP, 18% tris-glycine gels were also included) and electroblotted onto Hybond-C nitrocellulose membranes (Amersham Biosciences, Piscataway, NJ, USA). The membranes were then processed for 1 h at RT with 5% skim milk to block non-specific binding sites and incubated overnight at 4°C with various indicated primary antibodies. After rinsing in 1xTBST, membranes were further incubated with HRP-conjugated secondary antibodies for 1 h at RT, developed in ECL solution for 1-5 min and exposed onto X-films for 1-5 min. Antibodies including p-ERK, p-p38 and p-CaMKII were purchased from Cell Signaling Technology and used at a dilution of 1:1000 while CGRP (1:1000) was obtained from Peninsula Labs and nNOS (1:800) from Santa Cruz Biotechnology. For loading controls, membranes were incubated with a stripping buffer and reprobed with antibodies against ERK, p38 and CaMKII at a dilution of 1:1000 (Cell Signaling Technology) as well as beta-actin at a dilution of 1:5000 (Santa Cruz Biotechnology). Specific bands were captured using a Sierra Scientific MS-4030 video camera (Sierra Scientific, Sunnyvale, CA, USA)and quantified by densitometric analysis using MCID version 6 Elite imaging software (Imaging Research, St. Catharines, ON, Canada). The density of each target band was normalized with its corresponding internal loading control bands. The fold change was defined as the ratio of the normalized data of the treatment group relative to its control group. Data are expressed as mean ± SEM. Statistical significance was analyzed using one-way or two-way ANOVA, wherever appropriate followed by a SNK *post hoc *test for comparisons between groups.

## List of abbreviations used

7-NI: 7-Nitroindazole; CaMKII: calmodulin-dependent protein kinase II; CGRP: calcitonin gene-related peptide; CREB: cAMP response element-binding protein; DRG: dorsal root ganglion; ERK: extracellular signal-regulated protein kinase; NG: (4S)-N-(4-Amino-5[aminoethyl]aminopentyl)-N'-nitroguanidine; nNOS: neuronal nitric oxide synthase; NO: nitric oxide; PKA: protein kinase A; SCDH: spinal cord dorsal horn; sGC: soluble guanylate cyclase.

## Competing interests

The authors declare that they have no competing interests.

## Authors' contributions

ZW designed and carried out the experiment and drafted the manuscript. JGC contributed to the image analysis and data processing. RQ conceived and provided overall guidance to the study and contributed to the manuscript preparation. All authors read and approved the final manuscript.

## Supplementary Material

Additional file 1 **Additional figures 1 and 2**. The file contains two figures, additional Figures 1 and 2. Click here for file
