# Supplementary material for: On the possible role of ERK, p38 and CaMKII in the regulation of CGRP expression in morphine-tolerant rats
Source: Mol Pain. 2011 Sep 21;7:68. doi: 10.1186/1744-8069-7-68 (PMC3190348; doi:10.1186/1744-8069-7-68)
Supplement: Additional file 1 — Additional figures 1 and 2. The file contains two figures, additional Figures 1 and 2. [file 1744-8069-7-68-S1.DOC]

**Additional File 1**

**Figure Legends**

**Figure 1** Western blot analyses of the nNOS and CGRP levels in the spinal cord dorsal horn following a chronic treatment with various inhibitors alone. A 7-day treatment with PD98059 (PD, 10 μg), an MEK inhibitor, SB203580 (SB, 10 μg), a p38 inhibitor or KN93 (KN, 15 nmol), a CaMKII inhibitor did not change the expression of nNOS or CGRP when compared with saline (NS) group.

**Figure 2** Double immunofluorescence reveals that CaMKII is mostly enriched in NeuN-ir (the neuronal marker) cells (arrows), and not in GFAP- (green, the astroglial marker) or OX-42-ir (green, the microglia marker) cells. Scale bar, 20 μm.

**Figure 1**

**
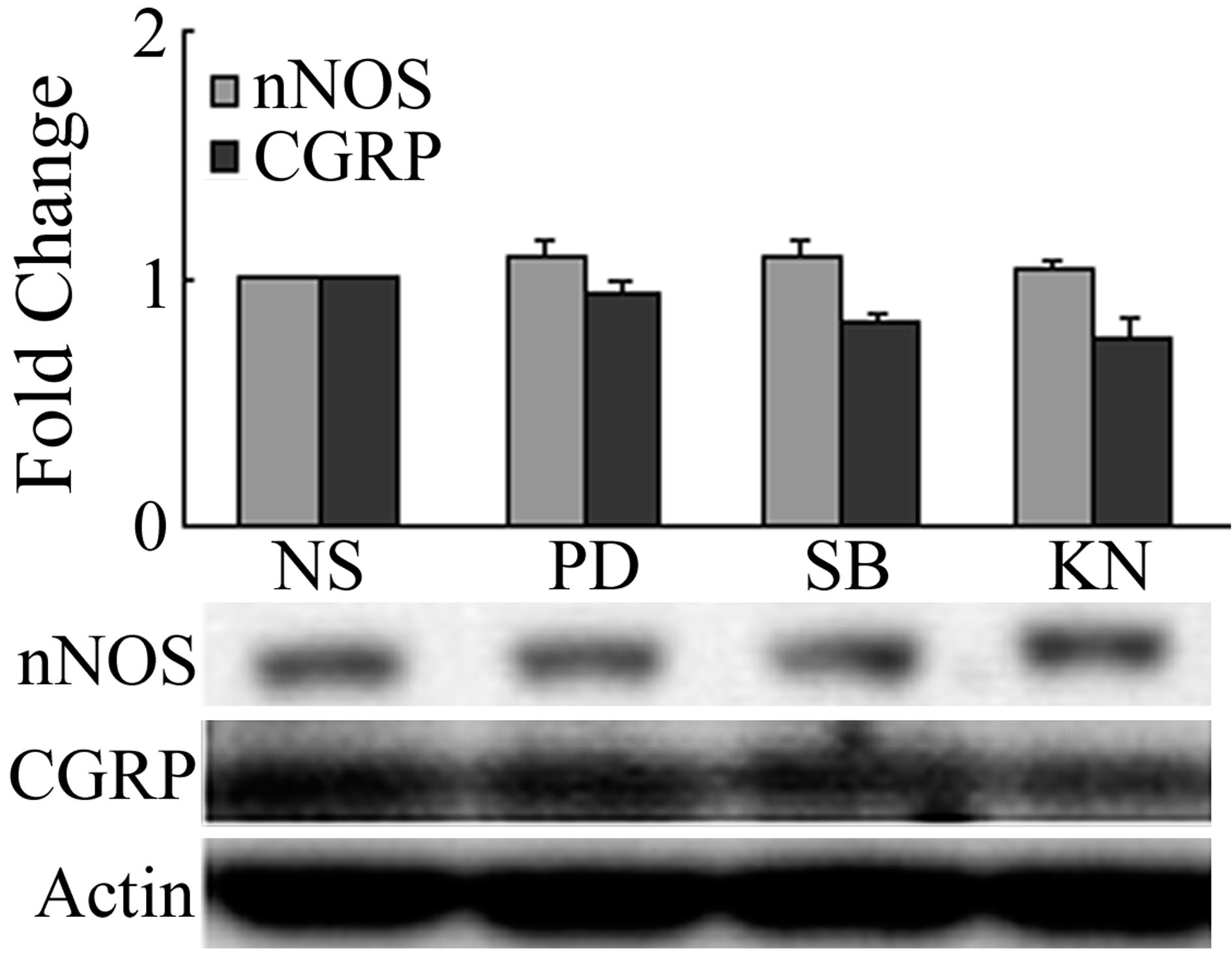
**

**Figure 2**

**
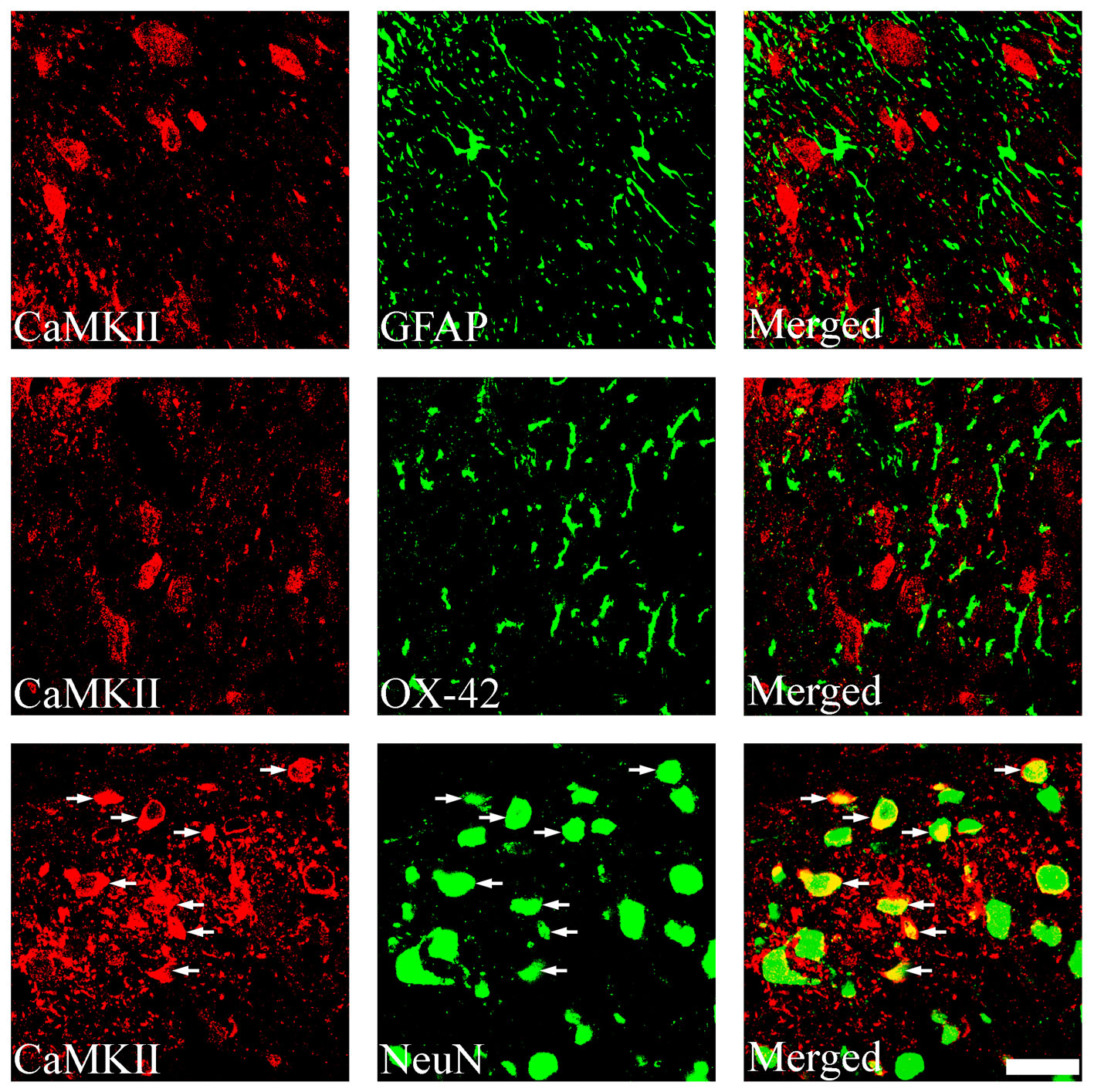
**
